# Analysis of the minimal specificity of caspase-2 and identification of Ac-VDTTD-AFC as a caspase-2-selective peptide substrate

**DOI:** 10.1042/BSR20140025

**Published:** 2014-03-25

**Authors:** Tanja Kitevska, Sarah J. Roberts, Delara Pantaki-Eimany, Sarah E. Boyd, Fiona L. Scott, Christine J. Hawkins

**Affiliations:** *Department of Biochemistry, La Trobe Institute for Molecular Science, La Trobe University, Bundoora 3086, Victoria, Australia; †Receptos, 10835 Road to the Cure, Suite 205, San Diego, CA 92121, U.S.A.

**Keywords:** apoptosis, caspase, cleavage, protease, Runx1, substrate, CED-3, cell-death determining 3, HEK-293T cells, human embryonic kidney cells expressing the large T-antigen of SV40 (simian virus 40), IPTG, isopropyl β-D-thiogalactoside, ONPG, *o*-nitrophenyl β-D-galactopyranoside

## Abstract

Caspase-2 is an evolutionarily conserved but enigmatic protease whose biological role remains poorly understood. To date, research into the functions of caspase-2 has been hampered by an absence of reagents that can distinguish its activity from that of the downstream apoptotic caspase, caspase-3. Identification of protein substrates of caspase-2 that are efficiently cleaved within cells may also provide clues to the role of this protease. We used a yeast-based transcriptional reporter system to define the minimal substrate specificity of caspase-2. The resulting profile enabled the identification of candidate novel caspase-2 substrates. Caspase-2 cleaved one of these proteins, the cancer-associated transcription factor Runx1, although with relatively low efficiency. A fluorogenic peptide was derived from the sequence most efficiently cleaved in the context of the transcriptional reporter. This peptide, Ac-VDTTD-AFC, was efficiently cleaved by purified caspase-2 and auto-activating caspase-2 in mammalian cells, and exhibited better selectivity for caspase-2 relative to caspase-3 than reagents that are currently available. We suggest that this reagent, used in parallel with the traditional caspase-3 substrate Ac-DEVD-AFC, will enable researchers to monitor caspase-2 activity in cell lysates and may assist in the determination of stimuli that activate caspase-2 *in vivo*.

## Abstract 2

The roles of the protease caspase-2 are currently poorly understood. Research into caspase-2 would benefit from more specific tools to monitor its activity. A more caspase-2-selective peptide, Ac-VDTTD-AFC, may help researchers to detect caspase-2 activity in cell extracts.

## INTRODUCTION

Caspases are cysteine proteases, most of which play well-defined roles in apoptosis and/or inflammation. However, despite being the second mammalian caspase to be identified, and the most evolutionarily conserved, caspase-2 remains a mysterious enzyme whose function and biological importance are still poorly understood. Like other caspases bearing large N-terminal interaction domains, caspase-2 can be activated by induced proximity following recruitment to intermolecular complexes [1] such as the PIDDosome, which also contains PIDD (p53-inducible protein with a death domain) and the adaptor protein RAIDD {RIP (receptor-interacting protein)-associated ICH-1 [ICE (interleukin-1β-converting enzyme)/CED-3 (cell-death determining 3) homologue 1] protein with a death domain}[2]. Accumulating evidence implies that alternative activating complexes may also exist [3–6]. The activity of caspase-2 can also be regulated by phosphorylation, which has been shown to suppress its activity during mitosis or nutrient abundance [7]. Caspase-2 has been reported to reside in nuclei, cytosol and golgi. Two groups detected activated caspase-2 in the cytoplasm following apoptotic stimuli, although one study suggested that the caspase was activated in the nucleus prior to export [8], whereas the other implied that activation occurred in the cytosol [9].

Caspase-2 seems not to play a unique role during development. Initial analyses of caspase-2-null mice only revealed a slight increase in oocytes and temporary boost in numbers of facial neurons [10]. Caspase-2 deficiency also led to exaggeration of ageing-related characteristics, seemingly due to increased oxidative damage [11,12]. Although caspase-2 knockout mice do not spontaneously develop cancers, more recent analyses of cancer-prone mice lacking this caspase have exposed a tumour-suppressor function. Fibroblasts lacking caspase-2 [13] or expressing an active site mutant [14] promoted oncogenic transformation *in vitro*. As well, caspase-2 loss enhanced lymphoma formation in mice bearing the Eμ-myc transgene [4,13] or lacking ATM (ataxia telangiectasia mutated) [15]. However, the anti-cancer potential of caspase-2 varied between experimental models. Caspase-2 deletion only slightly accelerated tumour development in mice expressing the c-neu oncogene in mammary cells [16] and failed to alter cancer incidence following irradiation or exposure to 3-methylcholanthrene [4]. Consistent with a tumour-suppressor function, caspase-2 deletion, mutation or down-regulation have been reported in some human cancers (reviewed by [17]).

Cell-based experiments have also been performed to help define the activity of caspase-2, yet these have yielded conflicting results. Like many proteases, caspase-2 can kill cells upon overexpression [18,19]. Some data suggested that it could trigger apoptotic signalling in response to heat shock, ER (endoplasmic reticulum) stress, death ligands or DNA damage [20–26], yet other studies disputed any involvement of caspase-2 in these apoptotic pathways [27–30]. Caspase-2 was recently implicated in the lethality of bacterial pore-forming toxins [5]. Other data suggest that caspase-2 may perform a cell-cycle checkpoint role. Prevention of phosphorylation of caspase-2 on residue Ser^340^ during mitosis led to its activation and stimulated apoptosis [31]. Cells lacking caspase-2 failed to arrest following irradiation [13], consistent with data showing that caspase-2 was required for robust P53-dependent repair of DNA damage [16,32]. Chk-1 inhibition coupled with γ-irradiation provoked apoptosis that was caspase-2-dependent, but P53-independent [33–34].

Some of the confusion regarding the function of caspase-2 may stem from researchers’ use of non-specific tools for monitoring its activity and inhibition. Talanian et al. [35] published in 1997 that caspase-2 could efficiently cleave peptides containing the sequence VDVAD (like Ac-VDVAD-pNA), but simultaneously reported that caspase-3 could cleave these peptides with similar kinetics. Subsequent studies have confirmed the lack of specificity associated VDVAD-based tools [29,36–38], yet many researchers continue to employ substrates and inhibitors derived from this peptide sequence as caspase-2-specific reagents. Our understanding of the roles played by caspase-2 would be greatly facilitated by development of more selective tools, especially probes that can monitor caspase-2 activity without detecting caspase-3 activity. Maillard et al. [38] created a caspase-2 inhibitor by chemically modifying the P2 position of the VDVAD sequence. Encouragingly, their best compound showed improved selectivity for caspase-2 relative to caspase-3, but unfortunately had lower affinity for caspase-2 than the parental VDVAD peptide. Nevertheless, that study highlights the feasibility of making peptide-based reagents that are specific for caspase-2.

It seems likely that identification of caspase-2 substrates may provide valuable clues to its biological role. A number of proteins have been reported to be cleaved by caspase-2 [39], but a few of those have been shown to be the specific substrates of caspase-2 (and not caspase-3), and hardly any have been shown to be cleaved by physiologically relevant concentrations of caspase-2 [40,41]. Apoptotic signalling by caspase-2 has been proposed to result from its processing of the pro-apoptotic Bcl-2 relative Bid. Initial results suggested that caspases-2 and -8 could cleave Bid with similar efficiency [42], but subsequent data suggested that the processing by caspase-8 was more efficient [43,44]. Caspase-7 was also weakly sensitive to caspase-2-mediated proteolysis [44,45], but it is not clear whether this contributes to any apoptotic activity of caspase-2. Caspase-2-mediated cleavage of Golgin-160 [46] could contribute to the destruction of the Golgi complex during apoptosis. The ability of caspase-2 to proteolytically disable MDM2 (murine double minute 2) provides a potential mechanism through which caspase-2 could provoke P53-dependent cell-cycle arrest or apoptosis, yet P53 was dispensable for caspase-2-mediated apoptosis following Chk1 inhibition and irradiation [33] and caspase-2-modulated P53 phosphorylation rather than its degradation following irradiation of fibroblasts [32]. Caspase-2 efficiently cleaved the transcription factor Cux1 [47]. Other caspases could also cleave Cux1, yet the nuclear localization of caspase-2 may give it unique access to this substrate. However, cleaved Cux1 promoted rather than prevented S phase entry [47], in contrast to the above-mentioned role attributed to caspase-2 in halting cell-cycle progression. A degradomics approach identified a number of proteins that were cleaved in lysates incubated with caspase-2 [41]. While most were also processed following incubation with other caspases, the translation initiation factor EIF4B was one of a minority which were more efficiently cleaved by caspase-2 [41]; however, the significance of this cleavage event remains to be defined.

We reasoned that elucidation of the biological role of caspase-2 would be facilitated by the development of more specific tools for monitoring its activity, and by identification of additional substrates. In this study, we defined the minimal (P5–P1′) specificity of caspase-2 using a yeast-based transcriptional reporter system. We used the specificity data yielded by that approach to develop a caspase-2-selective fluorogenic substrate, and to conduct bioinformatics screening for candidate protein substrates.

## MATERIALS AND METHODS

### Plasmids

The following plasmids have been previously described: pGALL-(*HIS3*)-caspase-2 [44], pGALL-(*TRP1*)-CD4-DETD’G-LexAB42, pGALL-(*TRP1*)-CD4-DETE’G-LexAB42, pGALL-(*TRP1*)-CD4-DETG’G-LexAB42, pGALL-(*TRP1*)-CD4-DETX’G-LexAB42, pGALL-(*TRP1*)-CD4-XXXD’G-LexAB42 [48]. Other reporter constructs were made using a method described earlier [48], by performing PCR with a cleavage site-specific 5′ primer and the common 3′ primer (1200; see list below), cutting the product with EcoRI and BglII and cloning into pGALL-(*TRP1*)-CD4-DETE-LexAB42 [48] cut with EcoRI and BglII. The 5′ primers used to make constructs containing the various cleavage sites were DVPD: 1620, DVPG: 1621, DTTD: 1616, DTTG: 1617, VDVAD: 1618, VDVAG: 1619, DETD’X (P1′ library): 1254, XDETD (P5 library): 1255. The 5′ portion of Bid was amplified with primers 702 and 1339 and cut with EcoRI and NheI. Mutant 3′ portions were amplified with primer 704 and either 1337 (DTTD) or 1338 (DTTG) and cut with NheI and BamHI. These fragments were cloned into pBluescriptII(SK+) cut EcoRI/BamHI as three-way ligations. These constructs and Bluescript plasmids bearing wild-type Bid and Bid^LQTG^ [44] were used as templates for PCR reactions with primers 702 and 1622. These products were cut with EcoRI and XhoI and cloned into pET23a–noT7 [49] for purification of C-terminally His_6_-tagged proteins. Bid^DVPD^–pET23a–noT7 and Bid^DVPA^–pET23a–noT7 were generated by amplifying the 3′ part of Bid using oligonucleotides 1188 and 1689 (DVPD) or 1690 (DVPA), cutting the product with NheI and XhoI and replacing the NheI/XhoI fragment of Bid^DTTA^–pET23a–noT7. Caspase-8^Δ1−216^ was amplified using primers 819 and 820, cut with NdeI and BamHI and cloned into pET15b (Novagen). Runx1B was amplified from pFlagCMV2-Runx1B (Addgene plasmid 12504, deposited by Dong-Er Zhang) with primers 1464 and 1420. The product was cut with EcoRI and XhoI then cloned into pET23a–noT7 cut EcoRI/XhoI. To create the Runx1^D99G^ mutant, the middle portion of the gene (AvrII–HindIII) was replaced with the corresponding region containing the P1 mutation, which was amplified using the mutagenic primer 1313. Auto-activating caspase-3 was amplified from pGALL-(*LEU2*)-revCaspase-3 [50] with primers 1634 and 1595. Auto-activating caspase-2 was amplified from pGALL-(*LEU2*)-rev-caspase-2 [44] with primers 1447 and 1595. Each product was cut with BamHI and XbaI and ligated into pEF-puro [51] cut with BamHI and XbaI.

### Oligonucleotides

Oligonucleotides used were: 702, 5′-GGAATTCGCCGCCAT-GGACTGTGAGGTCAACAACGG-3′; 704, 5′-CCGGATCCT-CAGTCCATCCCATTTCTGGC-3′; 819, 5′-GGAATTCCATA-TGAGTGAATCACAGACTTTGG-3′; 820, 5′-GCGGATCCT-CAATCAGAAGGGAAGACAAG-3′; 1188, 5′-GCTAGTTATT-GCTCAGCGG-3′; 1200, 5′-CCGCTCGAGCTAATCTCCACT-CAGCAAGAGGCTGGTATC-3′; 1254, 5′-CGGAATTCGATG-AGACGGATNNSATGAAAGCGTTAACGGCCAGGCAACA-AG-3′; 1255, 5′-CGGAATTCNNSGATGAGACGGATGGCAT-GAAAGCGTTAACGGCCAG-3′; 1313, 5′-GTGGCCCTAGG-GGATGTTCCAGGTGGCACTCTGGTCAC-3′; 1337, 5′-CCA-GTGCTAGCTCCCCAGTGGGAGGGCTACGATGAGGACA-CGACTGATGGCAACCGCAGCAGCCAC-3′; 1338, 5′-CCA-GTGCTAGCTCCCCAGTGGGAGGGCTACGATGAGGACA-CGACTGCTGGCAACCGCAGCAGCCAC-3′; 1339, 5′-CCA-CTGGGGAGCTAGCACTGGCAGCTCGTG-3′; 1420, 5′-G-ACCTCGAGGTAGGGCCTCCACACGGCCTC-3′; 1447, 5′-TGCAGATCTCCACCATGGACCAACAAGATGGAAAG-3′; 1464, 5′-AGGAATTCATATGCGTATCCCCGTAGATGCCAG-CACGAGCCGC-3′; 1553, 5′-GCCGACCTCGAGGTCCATC-CCATTTCTGGCTAAG-3′; 1595, 5′-CTTTATTATTTTTATTT-TATTGAGAGGGTGG-3′; 1616, 5′-CGGAATTCGACACAAC-AGACGGCATGAAAGCGTTAACGGCCAGGCAACAAG-3′; 1617, 5′-CGGAATTCGACACAACAGGCGGCATGAAAGC-GTTAACGGCCAGGCAACAAG-3′; 1618, 5′- CGGAATTCG-UAGACGUAGCCGACGGCATGAAAGCGTTAACGGCCA-GGCAACAAG-3′; 1619, 5′-CGGAATTCGUAGACGUAGC-CGGCGGCATGAAAGCGTTAACGGCCAGGCAACAAG-3′; 1620, 5′-CGGAATTCGACGUACCCGACGGCATGAAAGCG-TTAACGGCCAGGCAACAAG-3′; 1621, 5′-CGGAATTCGA-CGUACCCGGCGGCATGAAAGCGTTAACGGCCAGGCA-ACAAG-3′; 1622, 5′-CCGCTCGAGGTCCATCCCATTTCTG-GC-3′; 1634, 5′- GTCGGATCCACCATGATTGAGACAGAC-AGTGGTGTTG-3′; 1689, 5′- CCAGTGCTAGCTCCCCA-GTGGGAGGGCTACGATGAGGACGTGCCTGATGGCAAC-3′; and 1690, 5′- CCAGTGCTAGCTCCCCAGTGGGAGGGCT-ACGATGAGGACGTGCCTGCTGGCAACCGC-3′

### Yeast and bioinformatics techniques

Yeast methods including transformation, DNA extraction, library screening, Xgal and ONPG (*o*-nitrophenyl β-D-galactopyranoside) assays were performed as previously described [48,52]. Weighted P4–P2 transcriptional reporter data were used to generate a matrix model (Supplementary Table S1; available at http://www.bioscirep.org/bsr/034/bsr034e100add.htm). The Web-based PoPS program [53] was used to screen the National Center for Biotechnology Information (NCBI) protein database for potential substrates.

### Protein production

Caspases-2, -3 and -8 were purified using NiNTA resin (Qiagen) from BL21(DE3)pLysS bacteria (Novagen/Merck) transformed with pET23a–caspase-2^Δ1−149^ [44], pET23a–caspase-3 [54] or pET15b–caspase-8^Δ1−216^ plasmids. Induction conditions were: caspase-2, 0.4 mM IPTG (isopropyl β-D-thiogalactoside), 2 h 37°C; caspase-3, 0.2 mM IPTG, 3 h 30°C; caspase-8, 1 mM IPTG, 4 h 25°C. Caspases were active site-titrated using zVAD-fmk (Merck) [55]. ArcticExpress DE3 bacteria (Agilent Technologies) were transformed with the pET23a Bid expression plasmids described above, induced for 48 h at 10°C using 1 mM IPTG, then the His_6_-tagged Bid proteins were purified using NiNTA resin (Qiagen). Recombinant proteins were quantitated using the Bicinchoninic acid protein assay kit (Sigma). Runx1 proteins were synthesized using the TNT T7 quick coupled transcription/translation system (Promega) with non-radioactive methionine, using intact Runx1–pET23a plasmids as templates. The concentration of Runx1 proteins was determined by quantitative anti-His_6_ immunoblotting, using purified AcP35–His_6_ [56] as a standard.

### Caspase cleavage assays

Caspases were pre-activated for 30 min at 37°C in their preferred buffers [57], then diluted and mixed with fluorogenic peptide substrates. Fluorescence (excitation 410 nm, emission 500 nm) was measured every 10–60 s for up to 1 h using a FluoStar Galaxy (BMG Labtech). The maximal slope of each curve was calculated. A standard curve, using free AFC (Sigma), was used to convert fluorescence emissions into concentration of AFC produced during the cleavage reactions. Prism 5.0 software was used to determine *k*_cat_ and *K*_M_ from three to four independent experiments, as previously described [49]. Ac-DEVD-AFC and Ac-VDVAD-AFC were purchased from Enzo Life Sciences. The custom substrates Ac-VDTTD-AFC and Ac-VDVPD-AFC were synthesized by 21st Century Biochemicals. 150 nM Bid or 100 nM Runx1 proteins were incubated with specified concentrations of caspases in their preferred buffers for 1 h (Runx1) or 0.5 h (Bid variants) at 37°C, then subjected to SDS–PAGE and immunoblotting with anti-Bid (#AF860 R&D Systems) or anti-His_6_ antibodies (#A00186 GenScript). Secondary antibodies were anti-mouse-HRP (#A9044, Sigma) or anti-goat-HRP (#6300-05, Southern Biotech). The SuperSignal West Dura Substrate (Thermo Scientific) was used to detect signals and quantitation of sub-saturated exposures was performed using a Syngene G:Box and Image Lab 3.0 software (BioRad). Linear regression was used to determine the caspase concentration, which cleaved half of each protein. These data were used to calculate the second-order rate constant (*k*), as an estimate of cleavage efficiency (*k*_cat_/*K*_M_), using a published protocol [55].

### Cell culture

HEK-293T cells [human embryonic kidney cells expressing the large T-antigen of SV40 (simian virus 40)] were cultured, transfected, harvested and lysed as described [58] except that protease inhibitors were not used. Each lysate was mixed with the appropriate fluorogenic substrate (0.1 mM) in caspase buffer, and fluorescence was monitored as outlined above.

## RESULTS

**Figure 1:**
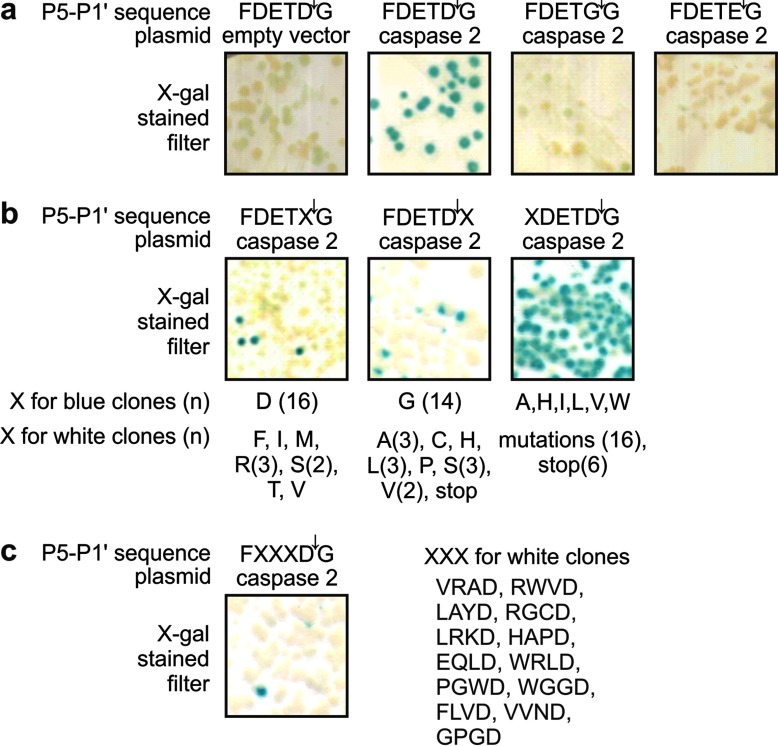
Profiling the minimal substrate specificity of caspase-2 using a transcriptional reporter system Yeast bearing a plasmid encoding a lexA-inducible β-galactosidase reporter gene were transformed with either an empty vector or caspase-2 expression plasmid, plus a vector encoding a fusion protein composed of a membrane anchor and LexA-B42 transcription factor domain separated by a cassette containing potentially cleavable sequences (‘P5–P1′ sequence’). Transformants were filter-lifted onto plates containing galactose, to induce expression of the caspase and fusion protein, then stained with Xgal to visualize any reporter gene activity that resulted from caspase-mediated cleavage of the fusion protein. In this assay, yeast colonies stain blue only when caspase-2 is expressed and is capable of cleaving ‘P5–P1′ sequence’. (**a**) The P1 specificity of caspase-2 was tested using fusion protein bearing sequences containing P1 aspartate, glutamate or glycine residues. (**b**) Yeast bearing the lexA-inducible β-galactosidase reporter plasmid and the caspase-2 expression plasmid were transformed with plasmid libraries encoding fusion proteins in which the transcription factor and membrane anchor were separated by the specified sequences including redundant residues occupying the P1, P1′ or P5 positions. Filters bearing transformant clones were stained with Xgal. Plasmids encoding the fusion proteins were extracted from indicated numbers of blue and white clones, and sequenced to identify caspase-2-cleavable and -uncleavable residues at each position. The residues encoded in the variable positions of these clones are listed. (**c**) Yeast bearing the lexA-inducible β-galactosidase reporter plasmid and the caspase-2 expression plasmid were transformed with a plasmid library encoding fusion proteins with redundant residues in positions P4–P2 of the linker domain. An Xgal-stained filter bearing induced transformant clones is shown. Plasmids encoding the fusion proteins were extracted from 13 white clones, and the P4–P1 sequences are shown. Analysis of the blue clones is presented in Figure 2.

We analysed the specificity of caspase-2 using a yeast-based transcriptional reporter system [52] that we had previously used to investigate the specificity of the nematode caspase CED-3 [48]. This system employs a chimeric protein composed of a membrane anchor derived from human CD4, a linker region containing potentially cleavable sequences, and the LexAB42 transcription factor. Cleavage results in release of the transcription factor from the plasma membrane, and transcription of the reporter gene lacZ. This can be visualized using the colorimetric β-galactosidase substrate Xgal or spectrophotometrically using ONPG. Initially we tested the control fusion proteins in which CD4 and LexAB42 domains were separated by the sequences DETD, DETE or DETG. Plasmids encoding these fusion proteins were transformed into yeast bearing a lacZ reporter plasmid and a caspase-2 expression plasmid (referred to hereafter as the ‘caspase-2 reporter’ strain) or yeast containing the lacZ reporter plus an empty vector. Transformants were stained with Xgal to reveal reporter gene activation. As expected, transformants co-expressing caspase-2 and the DETD fusion protein stained blue, while those bearing the DETE or DETG fusion proteins remained white (Figure 1a). This implied that caspase-2 could cleave after the sequence DETD in this molecular context in yeast, but that replacement of the P1 residue with glycine or glutamate prevented cleavage. To further define the specificity of caspase-2 in this context, we employed P1, P1′ and P5 libraries in which those residues were encoded by degenerate codons. Caspase-2 reporter strain yeast were transformed with these libraries, then transformants were stained with Xgal. DNA from blue and white clones was extracted and the linker regions were sequenced.

All of the blue clones from the P1 library bore aspartate residues (Figure 1b), confirming the expected requirement for aspartate in this position. Interestingly, glycine was present in the P1′ position in all of the blue clones from the P1′ library screen (Figure 1b). White clones from this screen contained various amino acids at this position including alanine, serine, valine and cysteine. The vast majority of the P5 library transformants stained blue with Xgal (Figure 1b). Sequencing of the reporter constructs isolated from the few white clones revealed that all contained stop codons, frameshift mutations or point mutations in the transcription factor domain. These data indicated that caspase-2 could tolerate all amino acids in the P5 position. Because this assay is not quantitative, these results do not rule out caspase-2 possessing subtle preferences for particular residues in this position, such as its previously reported preference for valine [59,60]

**Figure 2:**
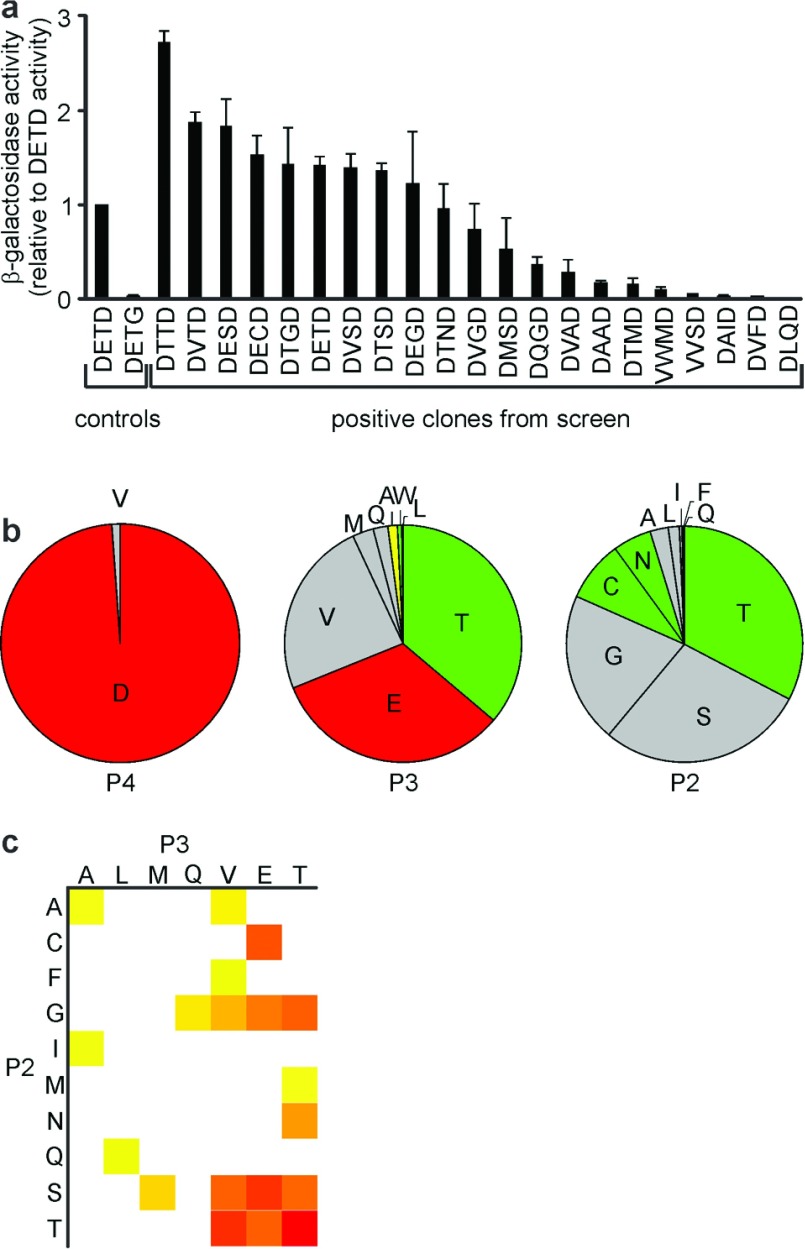
Defining the P4–P2 substrate specificity of caspase-2 using a transcriptional reporter system (**a**) Blue (positive) clones from the P4–P2 library screen described in Figure 1 were analysed in ONPG assays to quantitate reporter gene activity. The β-galactosidase activity of each positive clone is depicted, relative to the activity of clones expressing a fusion protein bearing the DETD control sequence. Error bars are S.E.M. from three to five independent assays. (**b**) The frequencies with which residues occupied P4, P3 and P2 positions of the fusion proteins are shown, weighted for the β-galactosidase activity of the clones containing each residue at each position. (**c**) A heat map shows combinations of P3 and P2 residues present in positive clones identified in the XXXD library. The colour intensity reflects the β-galactosidase activity of clones bearing each P3/P2 combination.

The caspase-2 reporter strain was also transformed with a library encoding random residues in the P4, P3 and P2 positions. Very few of these transformants stained blue with Xgal (Figure 1c), indicating that caspase-2 has strong preferences for subsites P4–P2. This result contrasted markedly with the promiscuous profile of CED-3 when screened with the same library [48]. The linker regions of the fusion proteins were sequenced from 21 blue clones (Figure 2) and 13 white clones (Figures 1c). Strikingly, all but one of the blue clones bore P4 aspartate residues. One clone had valine in this position, but quantitative ONPG assays revealed that the reporter gene activity for this clone was very weak (Figure 2a). Valine, glutamate and threonine were present in the P3 position of multiple positive clones that exhibited strong reporter gene activity. Ten amino acids were represented in the P2 site of positive clones, but serine and threonine were particularly prominent, as they were present in multiple positive clones that exhibited strong reporter gene activity (Figures 2b and 2c). Together, these data imply that caspase-2 most efficiently cleaves proteins when aspartate occupies the P4 position, P3 is valine, glutamate or threonine, P2 is serine or threonine, P1 is aspartate and P1′ is glycine.

**Figure 3:**
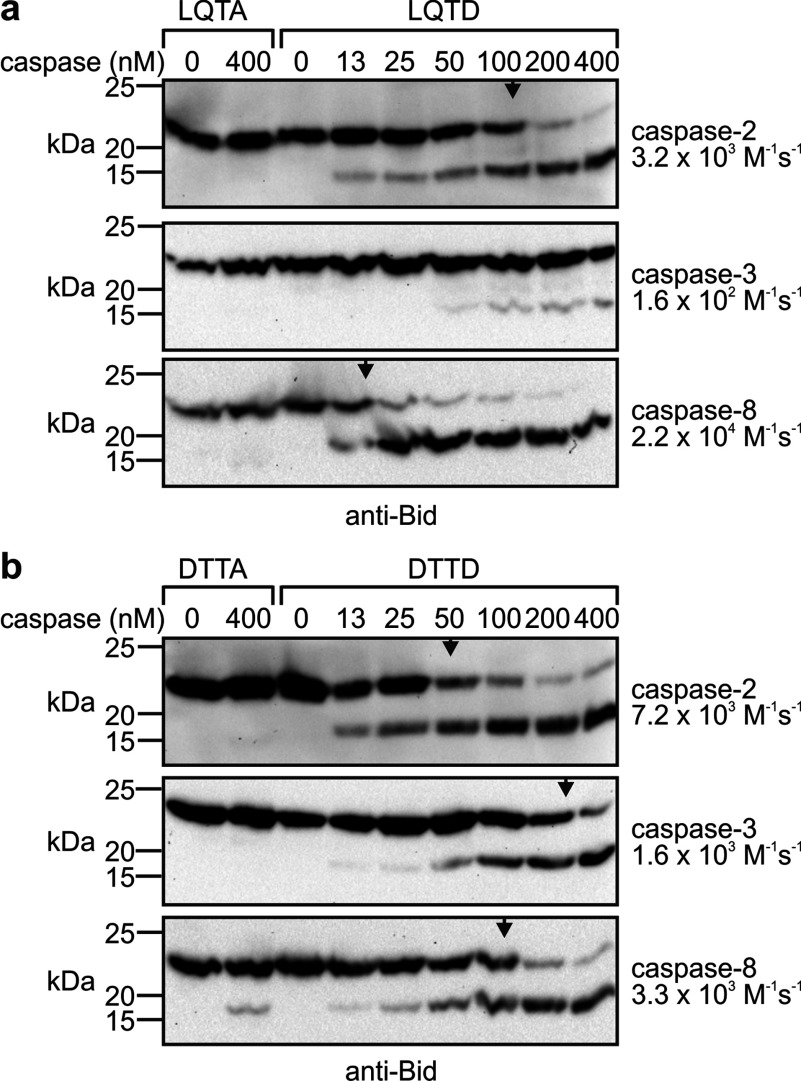
A Bid variant bearing the DTTD cleavage site is efficiently cleaved by caspase-2 Bid proteins bearing either (**a**) the native LQTD cleavage site or its P1 mutant (LQTA) or (**b**) the mutant sequence DTTD or its P1 mutant (DTTA) were incubated with the indicated concentrations of caspases-2, -3 or -8 and analysed by anti-Bid immunoblotting. Arrowheads denote the caspase concentrations at which half of the substrate was cleaved. The second-order rate constant, as an estimate of the cleavage efficiency, is stated to the right of each immunoblot.

The strongest reporter gene activity was detected in yeast bearing the cleavage sequence DTTD within the transcriptional reporter linker (Figures 2a and 2c). To directly investigate the efficiency with which caspase-2 cleaves this sequence in the context of a protein, we generated a Bid mutant in which the natural cleavage site LQTD^↓^G was replaced with the DTTD^↓^G sequence. P1 mutants of each were also created, in which aspartate was replaced with alanine. Proteins were purified from bacteria and incubated with a range of concentrations of caspases-2, -3 and -8 (Figure 3). As expected, wild-type Bid was processed about seven times more efficiently by caspase-8 than caspase-2, and was barely sensitive to proteolysis by even high concentrations of caspase-3. Caspase-2 cleaved the Bid^DTTD^ mutant about twice as efficiently as caspase-8 and about five times more efficiently than caspase-3. The corresponding P1 mutant, Bid^DTTA^, was resistant to cleavage by caspases-2 or -3. Unexpectedly, about 5% of this protein was cleaved by 400 nM caspase-8. In contrast, Bid^LQTA^ remained intact after treatment with each caspase. These results implied that caspase-8 could inefficiently cleave after the intended P4 aspartate (at the site YDED^↓^TTAGNR). This suggests that the estimate for caspase-8 cleavage at the DTTD^↓^G site may be somewhat overestimated.

**Figure 4:**
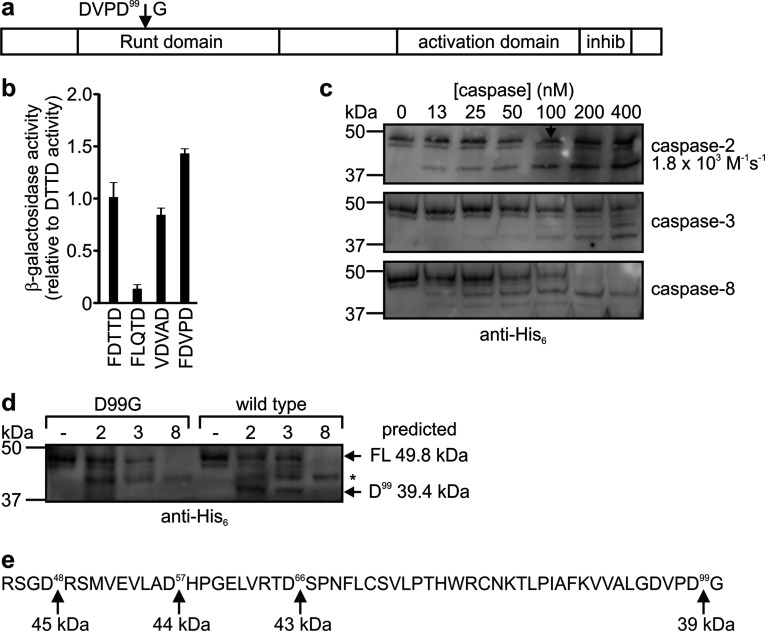
Runx1 is cleaved by caspase-2 at the site DVPD^99↓^G (**a**) The domain structure of the Runx1 protein and the predicted caspase-2 cleavage site are shown (Inhib; inhibitory domain). (**b**) Yeast bearing the lexA-inducible β-galactosidase reporter plasmid and the caspase-2 expression plasmid were transformed with plasmids encoding fusion proteins in which the transcription factor and membrane anchor were separated by the specified sequences. Induced clones were subjected to ONPG assays to quantitate reporter gene activity. Error bars are S.E.M. from the three independent assays. (**c**) *In vitro*-translated His-tagged Runx1 was incubated with the indicated concentrations of caspases-2, -3 or -8 and analysed by anti-His_6_ immunoblotting. (**d**) *In vitro*-translated Runx1 or its P1 mutant (‘D99G’) were incubated with 400 nM of caspases-2, -3 or -8 and analysed by anti-His_6_ immunoblotting. The migration of full-length Runx1 (FL) and the predicted cleavage product ‘D99’ are shown, with their predicted molecular weights. A second cleavage product is indicated by an asterisk. (**e**) The N-terminal portion of Runx1 contains the indicated aspartate residues, cleavage after which would yield products of the indicated sizes.

To identify novel candidate caspase-2 substrates, the PoPS program [53] was used to screen the human proteome for proteins that complied with a specificity matrix generated from the yeast transcriptional reporter data (Supplementary Figure S1). Twelve proteins were identified that contained potential caspase-2 cleavage sites within regions predicted to lie on the surface of the proteins (Supplementary Table S2 available at http://www.bioscirep.org/bsr/034/bsr034e100add.htm). In the light of data revealing a tumour-suppressor role for caspase-2, we were intrigued by the identification of the cancer-associated proteins Runx1 and 3 [61] as potential caspase-2 substrates. Caspase-2 was predicted to cleave Runx1 at the site DVPD^↓^G (Figure 4a). This candidate cleavage site was engineered into the transcriptional reporter fusion protein and caspase-2 reporter yeast transformed with this construct were tested for reporter gene activation. Robust β-galactosidase activity was observed, confirming that this sequence can be sensitive to caspase-2-mediated cleavage (Figure 4b). Caspase-2 could cleave *in vitro*-translated wild-type Runx1, but relatively high concentrations of the enzyme were required to achieve this proteolysis (Figure 4c). Caspase-2 cleaved Runx1 about half as efficiently as it cleaved wild-type Bid. Caspases-3 and -8 could also cleave Runx1, but the dominant product of these reactions was larger than that generated by caspase-2. Caspase-8 incubation led to a decrease in the total detectable Runx1 signal, suggesting that it may also cleave the protein near the tagged carboxyl terminus. Incubation of a P1 Runx1^DVPG^ mutant with caspases-2, -3 or -8 also yielded a larger product (Figure 4d). These data imply that Runx1 can be cleaved by caspases-2 and -3 at DVPD^99↓^G, but a distinct upstream site is also sensitive to proteolysis by caspases-2, -3 and -8 (Figure 4e). Additional mutagenesis could be used to define this upstream cleavage site and to determine whether proteolysis at one site affects sensitivity to cleavage elsewhere.

**Figure 5:**
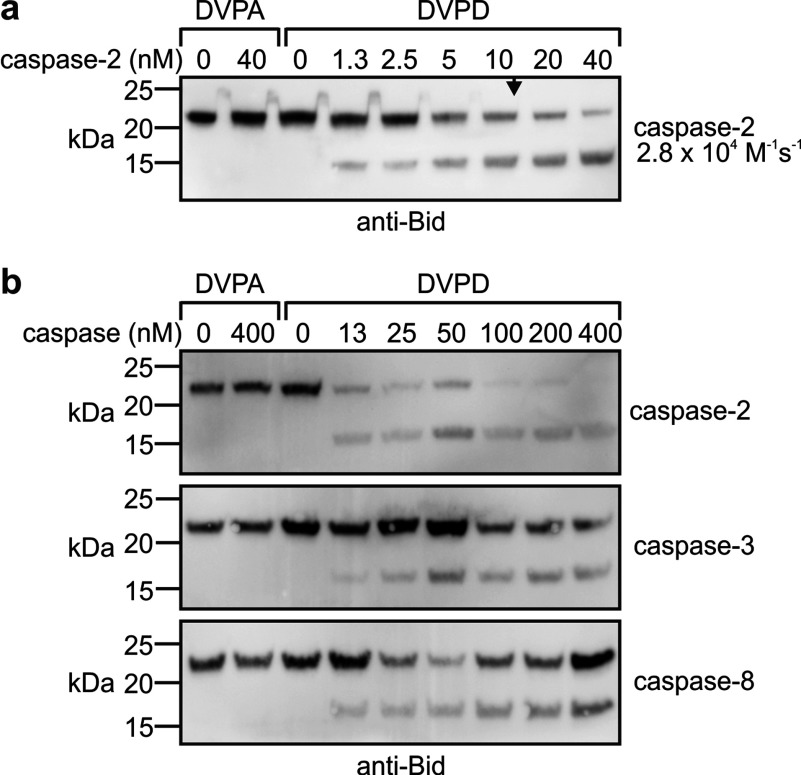
A Bid variant bearing the DVPD cleavage site is efficiently cleaved by caspase-2 Bid proteins bearing either a DVPD cleavage site or its P1 mutant (DVPA) were incubated with (**a**) 0–40 nM of caspase-2 or (**b**) 0–400 nM of caspases-2, -3 or -8 and analysed by anti-Bid immunoblotting. (**a**) The arrowhead denotes the caspase-2 concentration at which half of the substrate was cleaved. The second-order rate constant, as an estimate of the cleavage efficiency, is stated to the right of the immunoblot.

In the context of Runx1, the site DVPD^↓^G was cleaved more efficiently by caspase-2 than by caspases-3 or -8. To examine this cleavage specificity in another structural context, we generated Bid mutants in which the natural caspase-2/8 cleavage site (LQTD) was substituted with the sequences DVPD or DVPA. Low concentrations of caspase-2 cleaved Bid^DVPD^ (Figure 5a). Caspases-3 and -8 could also process this Bid variant, but with lower efficiency (Figure 5b).

**Figure 6:**
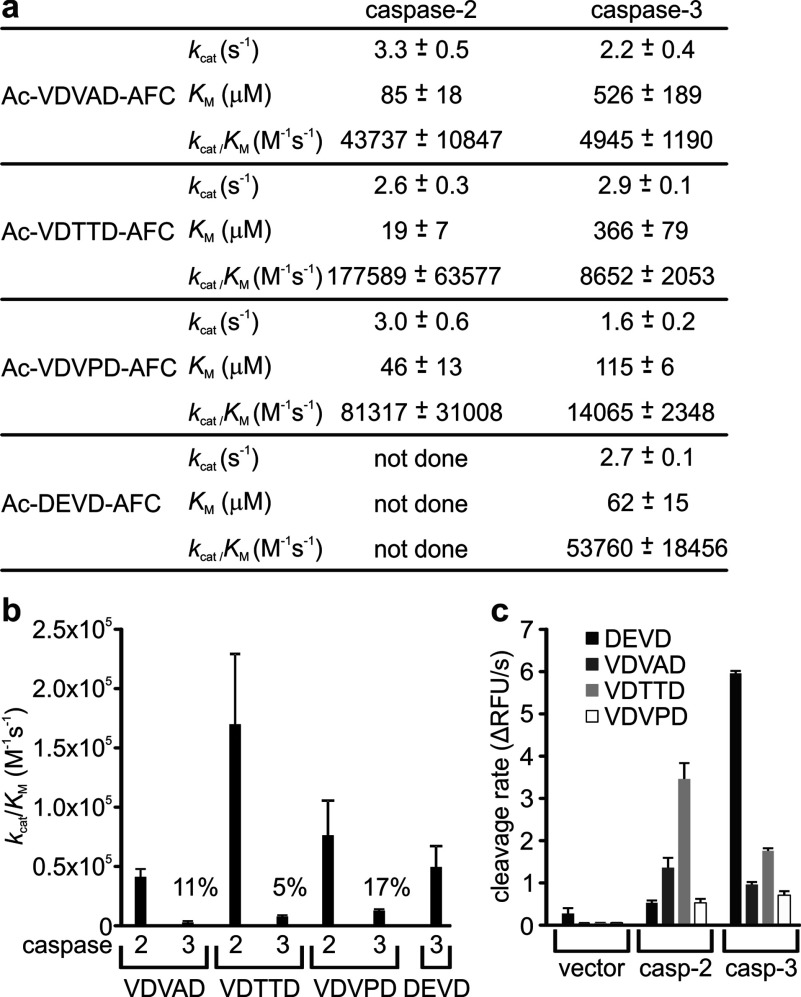
The fluorogenic substrate Ac-VDTTD-AFC exhibits enhanced caspase-2 sensitivity and specificity, relative to Ac-VDVAD-AFC (**a**) Active site-titrated caspases-2 or -3 were incubated with various concentrations of the indicated fluorogenic peptide substrates, and rates of increase in fluorescence were used to determine kinetics of cleavage. Data are presented as mean and S.E.M. from three to four independent assays for each caspase/substrate combination. (**b**) The efficiency (*k*_cat_/*K*_M_) for cleavage of each substrate by each caspase is graphed. The efficiency of cleavage of each substrate by caspase-3, relative to its cleavage by caspase-2, is denoted as a percentage. (**c**) HEK-293T cells were transiently transfected with plasmids encoding auto-activating forms of caspases-2 or -3, or empty vector. Lysates from these transfectants were mixed with the listed fluorogenic substrates and the rates of increase in fluorescence were used to measure cleavage efficiency. Data are presented as mean and S.E.M. from three independent transfections (RFU; relative fluorescence units).

As discussed above, many researchers use VDVAD-based reagents to monitor caspase-2 activity and inhibition in mammalian cells, however the efficient cleavage of this sequence by caspase-3 can seriously confound interpretation of such data. Given the enhanced selectivity of the DTTD and DVPD sequences for caspase-2 in the context of the Bid protein, we tested whether peptides bearing either of these sequences may constitute more selective tools for measuring caspase-2 activity than VDVAD-based peptides. We evaluated custom fluorogenic peptides in which these sequences were preceded by a P5 valine residue, as this was previously demonstrated to boost peptide cleavage by caspase-2 [35,60]. We compared cleavage by caspases-2 and -3 of these new peptides (Ac-VDTTD-AFC and Ac-VDVPD-AFC) with the classical caspase-2 substrate Ac-VDVAD-AFC and the traditional caspase-3 substrate Ac-DEVD-AFC (Figure 6a). Caspase-2 cleaved the Ac-VDTTD-AFC peptide four times as efficiently as Ac-VDVAD-AFC, but caspase-3 only cleaved it slightly more efficiently than Ac-VDVAD-AFC (Figure 6b). The peptide Ac-VDVPD-AFC, based on the Runx1 cleavage site, also exhibited enhanced sensitivity to caspase-2 relative to Ac-VDVAD-AFC. However, caspase-3 also proteolysed this substrate more efficiently than Ac-VDVAD-AFC, so Ac-VDVPD-AFC was less specific than Ac-VDVAD-AFC for caspase-2 relative to caspase-3 (Figure 6b).

We also investigated cleavage and specificity of these fluorogenic peptides in cell lysates. Auto-activating forms of caspases-2 or -3 were expressed in HEK-293T cells, and the lysates were compared for their abilities to cleave fluorogenic peptides containing the sequences DEVD, VDVAD, VDTTD and VDVPD. As expected from the assays using purified proteins, lysates from cells expressing active caspase-2 cleaved Ac-VDTTD-AFC more efficiently than the other peptides, and extracts from cells expressing active caspase-3 cleaved Ac-DEVD-AFC most efficiently (Figure 6c). Ac-VDTTD-AFC was proteolysed by lysates containing caspase-3, but at a slower rate than by lysates from cells expressing caspase-2. The classical ‘caspase-2’ fluorogenic substrate, Ac-VDVAD-AFC, was cleaved only slightly better by extracts from cells expressing caspase-2 than caspase-3 (Figure 6c). Thus, although the caspase-2 specificity of the Ac-VDTTD-AFC peptide in cell extracts was not absolute, this reagent was significantly more selective for caspase-2 than the commonly used Ac-VDVAD-AFC peptide.

## DISCUSSION

The yeast transcriptional reporter system demonstrated that efficient cleavage of a protein substrate by caspase-2 requires aspartate residues in the P4 and P1 positions, confirming earlier findings from peptide cleavage assays [35,62] and structural analyses [59]. These data contrasted slightly with results from a degradomics study, which identified a minority of caspase-2 substrates in which non-asparatate residues (chiefly glutamate) occupied the P4 position, although these substrates tended to be less sensitive to caspase-2 than proteins with aspartates at P4 [41]. In the context of the transcriptional reporter protein, caspase-2 absolutely required glycine in the P1′ position. Most of the caspase-2 substrates (including Bid, pro-caspase, golgin160 and EIF4B) contain glycine in P1′ position, but caspase-2 has also been reported to cleave proteins upstream of valine, serine, cysteine or alanine residues [41,47,63]. Presumably the molecular environment of the transcriptional reporter prevented larger amino acids from accessing the S1′ site of the enzyme. In the context of the transcriptional reporter, caspase-2 preferred valine, glutamate or threonine in the P3 position, broadly confirming the preferences at this position previously determined by positional library scanning [62]. Transcriptional reporter proteins bearing serine, threonine or glycine in P2 were efficiently cleaved in this study. This contrasted somewhat with Thornberry et al.'s positional library scanning data, which identified valine, isoleucine, threonine and proline to be preferred residues in this position [62]. Interestingly, the more sensitive caspase-2 substrates identified in Wejda et al.'s degradomics study tended to contain glutamate or arginine in P3 and proline or valine in P2 [41]. Although these discrepancies demonstrate that the molecular context outside of the immediate cleavage site can modulate sensitivity to cleavage by caspase-2, this study's transcriptional reporter assays proved useful in identifying sequences that were confirmed to be sensitive to caspase-2 proteolysis in other molecular environments. In particular, the P4–P1 sequence that was most efficiently cleaved by caspase-2 within the transcriptional reporter protein, DTTD^↓^, was also sensitive to proteolysis by caspase-2 within the context of a different protein (Bid) and a fluorogenic peptide (Ac-VDTTD-AFC).

Bioinformatic analyses, using the data from the transcriptional reporter system, predicted that caspase-2 could cleave Runx1. Runx1 plays a critical role in haematopoiesis, and Runx1 translocations or other mutations occur frequently in myeloid leukaemias [64], and less commonly in other cancer types [65]. Given that caspase-2 has been shown to reside in the nucleus and possess a tumour-suppressor function, the cancer-associated transcription factor Runx1 was an attractive candidate substrate for this protease. Caspase-2 was predicted to cleave Runx1 at the site DVPD^99↓^G, within the βB-C loop of the Runt DNA-binding domain (Figure 4A). This exposed loop is distant from the regions of the Runt domain that were shown to interact with the co-factor CBFβ and DNA [66–68] and hence may be accessible to proteases *in vivo*. This cleavage sequence partially matched the site (DVPD^↓^C) at which MDM-2 was reported to be cleaved by caspase-2, and less potently by caspase-3 [63]. *In vitro*-translated Runx1 was indeed cleaved by purified caspase-2, although with relatively poor efficiency. Runx1 was also cleaved, predominantly at other nearby positions, by caspases-3 and -8, illustrating the limitation of using a PoPS matrix that described the specificity of caspase-2 but did not impose penalties for sequences that were likely to be sensitive to cleavage by other proteases. Interestingly, caspase-2 cleaved the sequence DVPD much more efficiently when inserted into the Bid loop region than when embedded within Runx1. Indeed Bid^DVPD^ was processed by caspase-2 slightly more efficiently than caspase-8 cleaved wild-type Bid. These data therefore confirm the bioinformatics predictions that Runx1 could be cleaved by caspase-2 at DVPD^99↓^G. However, the inefficiency of this cleavage and the sensitivity of Runx1 to processing by other caspases argue against the notion that Runx1 is a caspase-2-specific substrate, which could account for the tumour-suppressor activity of this protease.

Research into caspase-2 biology has been hampered by a dearth of selective reagents capable of distinguishing between caspases-2 and -3. Peptides and inhibitors based on the sequence VDVAD are marketed as ‘caspase-2-specific’ but also react strongly with caspase-3. In the hope of generating more caspase-2-selective reagents, we evaluated custom fluorogenic peptides bearing sequences that this study revealed were more efficiently cleaved by caspase-2 than caspase-3. Ac-VDVPD-AFC, derived from the Runx1 cleavage site, was cleaved by caspase-2 slightly better than Ac-VDVAD, but its increased sensitivity to caspase-3-mediated proteolysis meant that it lacked the sought-after selectivity for caspase-2 over caspase-3. Confirming this lack of specificity, this substrate was cleaved at similar rates in cell lysates containing active caspases-2 or -3. Interestingly, Maillard et al. [38] found that the corresponding aldehyde inhibitors (Ac-VDVAD-CHO and Ac-VDVPD-CHO) inhibited caspases-2 and -3 with similar potencies.

More encouragingly, Ac-VDTTD-AFC emerged from this study as a useful substrate for caspase-2. Relative to Ac-VDVAD, this substrate was cleaved four times more efficiently by purified caspase-2 but only 1.7 times more efficiently by caspase-3. This selectivity was confirmed in assays using lysates of cells engineered to express active caspases-2 or -3. Lysates from cells expressing caspase-2 contained almost 14 times more VDTTDase activity than DEVDase activity (after background activity in the vector transfectant lysates was subtracted). In contrast, cells expressing active caspase-3 bore 3.3 times more DEVDase than VDTTDase activity. The field would benefit greatly from reagents that are absolutely caspase-2-specific. Unfortunately, Ac-VDTTD-AFC does not offer this degree of specificity. However, it does represent an improvement over the VDVAD-based tools that are commonly employed to monitor caspase-2 activity. Our data suggest that the combined use of DEVD- and VDTTD-based reagents could assist researchers to distinguish between caspase-2 and -3 activities in cell lysates, and thus help identify stimuli which activate caspase-2 in cells.

## Online data

Supplementary data

## References

[1] Mace P. D., Riedl S. J. (2010). Molecular cell death platforms and assemblies. Curr. Opin. Cell Biol..

[2] Tinel A., Tschopp J. (2004). The PIDDosome, a protein complex implicated in activation of caspase-2 in response to genotoxic stress. Science.

[3] Manzl C., Krumschnabel G., Bock F., Sohm B., Labi V., Baumgartner F., Logette E., Tschopp J., Villunger A. (2009). Caspase-2 activation in the absence of PIDDosome formation. J. Cell Biol..

[4] Manzl C., Peintner L., Krumschnabel G., Bock F., Labi V., Drach M., Newbold A., Johnstone R., Villunger A. (2012). PIDDosome-independent tumor suppression by caspase-2. Cell Death Differ..

[5] Imre G., Heering J., Takeda A. N., Husmann M., Thiede B., zu Heringdorf D. M., Green D. R., van der Goot F. G., Sinha B., Dotsch V., Rajalingam K. (2012). Caspase-2 is an initiator caspase responsible for pore-forming toxin-mediated apoptosis. EMBO J..

[6] Olsson M., Vakifahmetoglu H., Abruzzo P. M., Hogstrand K., Grandien A., Zhivotovsky B. (2009). DISC-mediated activation of caspase-2 in DNA damage-induced apoptosis. Oncogene.

[7] Buchakjian M. R., Kornbluth S. (2010). The engine driving the ship: metabolic steering of cell proliferation and death. Nat. Rev. Mol. Cell Biol..

[8] Tinnikov A. A., Samuels H. H. (2013). A novel cell lysis approach reveals that caspase-2 rapidly translocates from the nucleus to the cytoplasm in response to apoptotic stimuli. PLoS ONE.

[9] Bouchier-Hayes L., Oberst A., McStay G. P., Connell S., Tait S. W., Dillon C. P., Flanagan J. M., Beere H. M., Green D. R. (2009). Characterization of cytoplasmic caspase-2 activation by induced proximity. Mol. Cell.

[10] Bergeron L., Perez G. I., Macdonald G., Shi L., Sun Y., Jurisicova A., Varmuza S., Latham K. E., Flaws J. A., Salter J. C., Hara H., Moskowitz M. A., Li E., Greenberg A., Tilly J. L., Yuan J. (1998). Defects in regulation of apoptosis in caspase-2–deficient mice. Genes Dev..

[11] Zhang Y., Padalecki S. S., Chaudhuri A. R., De Waal E., Goins B. A., Grubbs B., Ikeno Y., Richardson A., Mundy G. R., Herman B. (2006). Caspase-2 deficiency enhances aging-related traits in mice. Mech. Ageing Dev..

[12] Shalini S., Dorstyn L., Wilson C., Puccini J., Ho L., Kumar S. (2012). Impaired antioxidant defence and accumulation of oxidative stress in caspase-2-deficient mice. Cell Death Differ..

[13] Ho L. H., Taylor R., Dorstyn L., Cakouros D., Bouillet P., Kumar S. (2009). A tumor suppressor function for caspase-2. Proc. Natl. Acad. Sci. U.S.A..

[14] Ren K., Lu J., Porollo A., Du C. (2012). Tumor-suppressing function of caspase-2 requires catalytic site Cys-320 and site Ser-139 in mice. J. Biol. Chem..

[15] Puccini J., Shalini S., Voss A. K., Gatei M., Wilson C. H., Hiwase D. K., Lavin M. F., Dorstyn L., Kumar S. (2013). Loss of caspase-2 augments lymphomagenesis and enhances genomic instability in Atm-deficient mice. Proc. Natl. Acad. Sci. U.S.A..

[16] Parsons M. J., McCormick L., Janke L., Howard A., Bouchier-Hayes L., Green D. R. (2013). Genetic deletion of caspase-2 accelerates MMTV/c-neu-driven mammary carcinogenesis in mice. Cell Death Differ..

[17] Puccini J., Dorstyn L., Kumar S. (2013). Caspase-2 as a tumour suppressor. Cell Death Differ..

[18] Kumar S., Kinoshita M., Noda M., Copeland N. G., Jenkins N. A. (1994). Induction of apoptosis by the mouse Nedd2 gene, which encodes a protein similar to the product of the Caenorhabditis elegans cell death gene CED-3 and the mammalian IL-1 beta-converting enzyme. Genes Dev..

[19] Wang L., Miura M., Bergeron L., Zhu H., Yuan J. (1994). ICH-1, an ICE/CED-3–related gene, encodes both positive and negative regulators of programmed cell death. Cell.

[20] Cao X., Bennett R. L., May W. S. (2008). c-Myc and caspase-2 are involved in activating Bax during cytotoxic drug-induced apoptosis. J. Biol. Chem..

[21] Ho L. H., Read S. H., Dorstyn L., Lambrusco L., Kumar S. (2008). Caspase-2 is required for cell death induced by cytoskeletal disruption. Oncogene.

[22] Upton J. P., Austgen K., Nishino M., Coakley K. M., Hagen A., Han D., Papa F. R., Oakes S. A. (2008). Caspase-2 cleavage of BID is a critical apoptotic signal downstream of endoplasmic reticulum stress. Mol. Cell Biol..

[23] Mhaidat N. M., Wang Y., Kiejda K. A., Zhang X. D., Hersey P. (2007). Docetaxel-induced apoptosis in melanoma cells is dependent on activation of caspase-2. Mol. Cancer Ther..

[24] Gao Z., Shao Y., Jiang X. (2005). Essential roles of the Bcl-2 family of proteins in caspase-2–induced apoptosis. J. Biol. Chem..

[25] Panaretakis T., Laane E., Pokrovskaja K., Bjorklund A. C., Moustakas A., Zhivotovsky B., Heyman M., Shoshan M. C., Grander D. (2005). Doxorubicin requires the sequential activation of caspase-2, protein kinase Cdelta, and c-Jun NH2–terminal kinase to induce apoptosis. Mol. Biol. Cell.

[26] Lin C. F., Chen C. L., Chang W. T., Jan M. S., Hsu L. J., Wu R. H., Tang M. J., Chang W. C., Lin Y. S. (2004). Sequential caspase-2 and caspase-8 activation upstream of mitochondria during ceramideand etoposide-induced apoptosis. J. Biol. Chem..

[27] Shelton S. N., Dillard C. D., Robertson J. D. (2010). Activation of caspase-9, but not caspase-2 or caspase-8, is essential for heat-induced apoptosis in Jurkat cells. J. Biol. Chem..

[28] O’Reilly L. A., Ekert P., Harvey N., Marsden V., Cullen L., Vaux D. L., Hacker G., Magnusson C., Pakusch M., Cecconi F. (2002). Caspase-2 is not required for thymocyte or neuronal apoptosis even though cleavage of caspase-2 is dependent on both Apaf-1 and caspase-9. Cell Death Differ..

[29] Delgado M. E., Olsson M., Lincoln F. A., Zhivotovsky B., Rehm M. (2013). Determining the contributions of caspase-2, caspase-8 and effector caspases to intracellular VDVADase activities during apoptosis initiation and execution. Biochim. Biophys. Acta.

[30] Sandow J. J., Dorstyn L., O’Reilly L. A., Tailler M., Kumar S., Strasser A., Ekert P. G. (2013). ER stress does not cause upregulation and activation of caspase-2 to initiate apoptosis. Cell Death Differ..

[31] Andersen J. L., Johnson C. E., Freel C. D., Parrish A. B., Day J. L., Buchakjian M. R., Nutt L. K., Thompson J. W., Moseley M. A., Kornbluth S. (2009). Restraint of apoptosis during mitosis through interdomain phosphorylation of caspase-2. EMBO J..

[32] Dorstyn L., Puccini J., Wilson C. H., Shalini S., Nicola M., Moore S., Kumar S. (2012). Caspase-2 deficiency promotes aberrant DNA-damage response and genetic instability. Cell Death Differ..

[33] Sidi S., Sanda T., Kennedy R. D., Hagen A. T., Jette C. A., Hoffmans R., Pascual J., Imamura S., Kishi S., Amatruda J. F. (2008). Chk1 suppresses a caspase-2 apoptotic response to DNA damage that bypasses p53, Bcl-2, and caspase-3. Cell.

[34] Ando K., Kernan J. L., Liu P. H., Sanda T., Logette E., Tschopp J., Look A. T., Wang J., Bouchier-Hayes L., Sidi S. (2012). PIDD death-domain phosphorylation by ATM controls prodeath versus prosurvival PIDDosome signaling. Mol. Cell.

[35] Talanian R. V., Quinlan C., Trautz S., Hackett M. C., Mankovich J. A., Banach D., Ghayur T., Brady K. D., Wong W. W. (1997). Substrate specificities of caspase family proteases. J. Biol. Chem..

[36] Pereira N. A., Song Z. (2008). Some commonly used caspase substrates and inhibitors lack the specificity required to monitor individual caspase activity. Biochem. Biophys. Res. Commun..

[37] McStay G. P., Salvesen G. S., Green D. R. (2007). Overlapping cleavage motif selectivity of caspases: implications for analysis of apoptotic pathways. Cell Death Differ..

[38] Maillard M. C., Brookfield F. A., Courtney S. M., Eustache F. M., Gemkow M. J., Handel R. K., Johnson L. C., Johnson P. D., Kerry M. A., Krieger F. (2011). Exploiting differences in caspase-2 and -3 S(2) subsites for selectivity: structure-based design, solid-phase synthesis and *in vitro* activity of novel substrate-based caspase-2 inhibitors. Bioorg. Med. Chem..

[39] Aksenova V. I., Bylino O. V., Zhivotovskii B. D., Lavrik I. N. (2013). Caspase-2: what do we know today?. Mol. Biol. (Mosk)..

[40] Kitevska T., Spencer D. M., Hawkins C. J. (2009). Caspase-2: controversial killer or checkpoint controller?. Apoptosis.

[41] Wejda M., Impens F., Takahashi N., Van Damme P., Gevaert K., Vandenabeele P. (2012). Degradomics reveals that cleavage specificity profiles of caspase-2 and effector caspases are alike. J. Biol. Chem..

[42] Guo Y., Srinivasula S. M., Druilhe A., Fernandes-Alnemri T., Alnemri E. S. (2002). Caspase-2 induces apoptosis by releasing proapoptotic proteins from mitochondria. J. Biol. Chem..

[43] Bonzon C., Bouchier-Hayes L., Pagliari L. J., Green D. R., Newmeyer D. D. (2006). Caspase-2–induced apoptosis requires Bid cleavage: a physiological role for bid in heat shock-induced death. Mol. Biol. Cell.

[44] Ho P. K., Jabbour A. M., Ekert P. G., Hawkins C. J. (2005). Caspase-2 is resistant to inhibition by inhibitor of apoptosis proteins (IAPs) and can activate caspase-7. FEBS J..

[45] Karki P., Dahal G. R., Shin S. Y., Lee J. S., Cho B., Park I. S. (2008). Efficient cleavage of Bid and procaspase-7 by caspase-2 at lower pH. Protein Pept. Lett..

[46] Mancini M., Machamer C. E., Roy S., Nicholson D. W., Thornberry N. A., Casciola-Rosen L. A., Rosen A. (2000). Caspase-2 is localized at the Golgi complex and cleaves golgin-160 during apoptosis. J. Cell Biol..

[47] Truscott M., Denault J. B., Goulet B., Leduy L., Salvesen G. S., Nepveu A. (2007). Carboxyl-terminal proteolytic processing of CUX1 by a caspase enables transcriptional activation in proliferating cells. J. Biol. Chem..

[48] Westein S. J., Scott F. L., Hawkins C. J. (2008). Analysis of the minimal specificity of CED-3 using a yeast transcriptional reporter system. Biochim. Biophys. Acta.

[49] Huang N., Civciristov S., Hawkins C. J., Clem R. J. (2013). SfDronc, an initiator caspase involved in apoptosis in the fall armyworm *Spodoptera frugiperda*. Insect Biochem. Mol. Biol..

[50] Hawkins C. J., Silke J., Verhagen A. M., Foster R., Ekert P. G., Ashley D. M. (2001). Analysis of candidate antagonists of IAP-mediated caspase inhibition using yeast reconstituted with the mammalian Apaf-1-activated apoptosis mechanism. Apoptosis.

[51] Hawkins C. J., Uren A. G., Hacker G., Medcalf R. L., Vaux D. L. (1996). Inhibition of interleukin 1–beta-converting enzyme-mediated apoptosis of mammalian cells by baculovirus IAP. Proc. Natl. Acad. Sci. U.S.A..

[52] Hawkins C. J., Wang S. L., Hay B. A. (2000). Monitoring activity of caspases and their regulators in yeast *Saccharomyces cerevisiae*. Methods Enzymol..

[53] Boyd S. E., Banda M. G. d.l., Pike R. N., Whisstock J. C., Rudy G. B. (2004). PoPS: a computational tool for modeling and predicting protease specificity. Proceedings of the IEEE Computer Society Bioinformatics Conference.

[54] Brand I. L., Green M. M., Civciristov S., Pantaki-Eimany D., George C., Gort T. R., Huang N., Clem R. J., Hawkins C. J. (2011). Functional and biochemical characterization of the baculovirus caspase inhibitor MaviP35. Cell Death Dis..

[55] Stennicke H. R., Salvesen G. S. (2000). Caspase assays. Methods Enzymol..

[56] Jabbour A. M., Ho P., Puryer M. A., Ashley D. M., Ekert P. G., Hawkins C. J. (2004). The *Caenorhabditis elegans* CED-9 protein does not directly inhibit the caspase CED-3, *in vitro* nor in yeast. Cell Death Differ..

[57] Garcia-Calvo M., Peterson E. P., Rasper D. M., Vaillancourt J. P., Zamboni R., Nicholson D. W., Thornberry N. A. (1999). Purification and catalytic properties of human caspase family members. Cell Death Differ..

[58] Beaumont T. E., Shekhar T. M., Kaur L., Pantaki-Eimany D., Kvansakul M., Hawkins C. J. (2013). Yeast techniques for modeling drugs targeting Bcl-2 and caspase family members. Cell Death Dis..

[59] Schweizer A., Briand C., Grutter M. G. (2003). Crystal structure of caspase-2, apical initiator of the intrinsic apoptotic pathway. J. Biol. Chem..

[60] Tang Y., Wells J. A., Arkin M. R. (2011). Structural and enzymatic insights into caspase-2 protein substrate recognition and catalysis. J. Biol. Chem..

[61] Ito Y. (2004). Oncogenic potential of the RUNX gene family: ‘overview’. Oncogene.

[62] Thornberry N., Rano T., Peterson E., Rasper D., Timkey T., Garcia-Calvo M., Houtzager V., Nordstrom P., Roy S., Vaillancourt J. (1997). A combinatorial approach defines specificities of members of the caspase family and granzyme B. Functional relationships established for key mediators of apoptosis. J. Biol. Chem..

[63] Oliver T. G., Meylan E., Chang G. P., Xue W., Burke J. R., Humpton T. J., Hubbard D., Bhutkar A., Jacks T. (2011). Caspase-2–mediated cleavage of Mdm2 creates a p53-induced positive feedback loop. Mol. Cell.

[64] Ichikawa M., Yoshimi A., Nakagawa M., Nishimoto N., Watanabe-Okochi N., Kurokawa M. (2013). A role for RUNX1 in hematopoiesis and myeloid leukemia. Int. J. Hematol..

[65] Chimge N. O., Frenkel B. (2013). The RUNX family in breast cancer: relationships with estrogen signaling. Oncogene.

[66] Tahirov T. H., Inoue-Bungo T., Morii H., Fujikawa A., Sasaki M., Kimura K., Shiina M., Sato K., Kumasaka T., Yamamoto M. (2001). Structural analyses of DNA recognition by the AML1/Runx-1 Runt domain and its allosteric control by CBFbeta. Cell.

[67] Backstrom S., Wolf-Watz M., Grundstrom C., Hard T., Grundstrom T., Sauer U. H. (2002). The RUNX1 Runt domain at 1.25A resolution: a structural switch and specifically bound chloride ions modulate DNA binding. J. Mol. Biol..

[68] Bravo J., Li Z., Speck N. A., Warren A. J. (2001). The leukemia-associated AML1 (Runx1)–CBF beta complex functions as a DNA-induced molecular clamp. Nat. Struct. Biol..

