# Supplementary material for: Analysis of the minimal specificity of caspase-2 and identification of Ac-VDTTD-AFC as a caspase-2-selective peptide substrate
Source: Biosci Rep. 2014 Mar 25;34(2):e00100. doi: 10.1042/BSR20140025 (PMC3966047; doi:10.1042/BSR20140025)
Supplement: Supplementary data [file bsr034e100add.pdf]

Biosci. Rep. (2014) / **34** / art:e00100 / doi 10.1042/BSR20140025

OPEN ACCESS

Original Paper

Bioscience Reports www.bioscirep.org

SUPPLEMENTARY DATA

Analysis of the minimal specificity of caspase-2 and identification of Ac-VDTTD-AFC as a caspase-2-selective peptide substrate

Tanja KITEVSKA*, Sarah J. ROBERTS*, Delara PANTAKI-EIMANY*, Sarah E. BOYD*[1], Fiona L. SCOTT† and Christine J. HAWKINS*[2]

*Department of Biochemistry, La Trobe Institute for Molecular Science, La Trobe University, Bundoora 3086, Victoria, Australia
†Receptos, 10835 Road to the Cure, Suite 205, San Diego, CA 92121, U.S.A.

Table S1 PoPS model for database screening
The frequencies of P4–P1′ residues from positive clones identified during transcriptional reporter screens (weighted based on β-galactosidase activity for P4–P2 residues) were assigned values from 0 to 5. Unrepresented residues were allocated a value of 0. PoPS was used to screen the predicted human proteome for potential substrates based on this model. Proteins were excluded from consideration if structural information predicted that at least three residues were buried or if less than three of the five residues were likely to be located in unstructured regions.

Subsites	S4	S3	S2	S1	S1′
Ala	0	0.065	0.12486	0	0
Arg	0	0	0	0	0
Asn	0	0	0.2633	0	0
Asp	4.945	0	0	5	0
Cys	0	0	0.4180	0	0
Gln	0	0.10043	0.00814	0	0
Glu	0	1.63	0	0	0
Gly	0	0	1.02877	0	5
His	0	0	0	0	0
Ile	0	0	0.0190	0	0
Leu	0	0.008	0.0760	0	0
Lys	0	0	0	0	0
Met	0	0.14	0	0	0
Phe	0	0	0.00814	0	0
Pro	0	0	0	0	0
Ser	0	0	1.42	0	0
Thr	0	1.807	1.63409	0	0
Trp	0	0.029	0	0	0
Tyr	0	0	0	0	0
Val	0.055	1.207	0	0	0

[1] Present address: School of Mathematical Sciences, Monash University, Clayton, Victoria 3800, Australia
[2] To whom correspondence should be addressed (email c.hawkins@latrobe.edu.au).

© 2014 The Author(s) This is an Open Access article distributed under the terms of the Creative Commons Attribution Licence (CC-BY) (http://creativecommons.org/licenses/by/3.0/) which permits unrestricted use, distribution and reproduction in any medium, provided the original work is properly cited.

Table S2 Candidate substrates identified by PoPS
High-ranking caspase-2 substrates are listed, which received PoPS scores above 15.

Score	Name	Description	Accession	Length	Predicted cleavage site
16.57	GRM1	Metabotropic glutamate receptor 1	NP_000829	1194	DEKD$^{135\downarrow}$G
16.23	NY-BR-1	Breast cancer autoantigen	NP_443723	1341	DVLD$^{47\downarrow}$G
16.17	EML3	Microtubule-binding protein	NP_694997	889	DVID$^{650\downarrow}$G
16.15	Runx1	Runt-related transcription factor; acute myeloid leukaemia gene, isoform b (also c)	NP_001001890	453	DVPD$^{99\downarrow}$G
16.15	Runx3	Runt-related transcription factor, tumor suppressor	NP_004341	415	DVPD$^{103\downarrow}$G
16.15	GFM2	Mitochondrial elongation factor isoform 1 (also 2 and 3)	NP_115756	779	DVDD$^{105\downarrow}$G
15.17	MTHFD1L	Mitochondrial C1-tetrahydrofolate synthase	NP_056255	978	DQAD$^{411\downarrow}$G
15.07	FAT1	Protocadherin Fat 1 precursor	NP_005236	4590	DDAD$^{3365\downarrow}$G
15.05	CASP2	Caspase 2	NP_116764	452	DQQD$^{333\downarrow}$G
15.01	WDR3	WD repeat-containing protein implicated in ribosomal subunit synthesis	NP_006775	943	DAHD$^{496\downarrow}$G
15.01	CSK	c-src tyrosine kinase	NP_004374	450	DAPD$^{409\downarrow}$G
15.01	ALDH1L2	Mitochondrial 10-formyltetrahydrofolate dehydrogenase precursor	NP_001029345	923	DADD$^{456\downarrow}$G

Received 5 February 2014/12 February 2014; accepted 13 February 2014

Published as Immediate Publication 17 February 2014, doi 10.1042/BSR20140025

© 2014 The Author(s) This is an Open Access article distributed under the terms of the Creative Commons Attribution Licence (CC-BY) (http://creativecommons.org/licenses/by/3.0/) which permits unrestricted use, distribution and reproduction in any medium, provided the original work is properly cited.
